# The outcomes of calcium silicate cement putty apical plugs in traumatised permanent maxillary teeth in paediatric patients: a retrospective evaluation

**DOI:** 10.1038/s41405-025-00317-9

**Published:** 2025-04-16

**Authors:** Dariusz Kasperek, Samantha Beattie, Nicholas Longridge, Fadi Jarad, Sondos Albadri

**Affiliations:** 1 ACF Specialty Registrar / DDSc Endodontics Trainee, Institute of Life Course and Medical Science, University of Liverpool, Liverpool, UK; 2 Dental Core Trainee, Institute of Life Course and Medical Science, University of Liverpool, Liverpool, UK; 3 Senior Clinical Lecturer and Honorary Consultant in Endodontics, Department of Restorative Dentistry, University of Liverpool, Liverpool, UK; 4 Professor and Honorary Consultant in Restorative Dentistry, Institute of Life Course and Medical Science, University of Liverpool, Liverpool, UK; 5 Professor in Paediatric Dentistry, Institute of Life Course and Medical Science, University of Liverpool, Liverpool, UK

**Keywords:** Root canal treatment, Endodontic instruments

## Abstract

**Aims:**

To evaluate the clinical and radiographic outcomes of root canal treatment of traumatised necrotic permanent maxillary teeth in paediatric patients.

**Methods:**

A retrospective analysis of clinical and radiographic records of patients aged 16 or under, who underwent apexification of permanent maxillary teeth, between 2016 and 2023, using TotalFill BC Putty was conducted. Success was assessed radiographically using periapical radiographs and clinically, against the European Society of Endodontology quality guideline consensus report.

**Results:**

A total of 66 teeth from 57 patients were included, with the mean age of 10 years old ( ± 2.1). The mean follow-up time was 14 months ( ± 12.2) with the average number of 3.5 visits ( ± 1.8). Success outcome at latest review was favourable in 48.5% (*n* = 32), uncertain in 36.4% (*n* = 24) and unfavourable in 15.1% (*n* = 10) of cases. In 84.9% (*n* = 56) of cases, there was evidence of complete or partial radiographic healing.

**Conclusions:**

Within the limits of this service evaluation, TotalFill BC Putty showed favourable outcomes in managing necrotic maxillary permanent teeth in children, demonstrating success rates comparable to MTA, suggesting that it may be a viable alternative for apexification in this patient cohort.

## Introduction

Dental trauma is highly prevalent, with approximately 20% of children and adolescents experiencing trauma to their permanent dentition at some point in their lives. The aetiology of traumatic dental injuries (TDI) is multifaceted and includes falls, sports-related injuries, traffic accidents, and assaults [1].

The consequences of TDI can be profound. Beyond immediate pain and functional/aesthetic concerns, there’s the potential for pulpal necrosis, resorption and ankylosis processes that can have life-time consequences for patients [2]. These pathological processes can additionally arrest root development and maintain an open apex which can present unique endodontic challenges. Historically, the management of such teeth included the process of apexification using non-setting calcium hydroxide, aiming to induce an apical hard tissue barrier [3]. Due to the extended treatment time and a possible increased risk of root fractures [4], an alternative approach of apexification with Mineral Trioxide Aggregate (MTA) was suggested [5]. MTA possesses desirable properties, including biocompatibility, antibacterial effects, and sealing ability [6]. Consequently, it has become widely favoured for use in endodontic procedures. However, due to MTA’s long setting time, unpredictable handling and high rates of tooth discolouration, newer hydraulic calcium silicate-based (HCSB) materials have been developed. These include dentine replacements such as Biodentine (Septodont, Saint-Maur-des-Fossès, France), root canal sealers, pastes and root repair material putties such as TotalFill BC Putty (FKG Dentaire, Switzerland). Their properties, include high alkalinity, which contributes to their anti-bacterial activity, as well as notable biocompatibility and osteogenic potential [7]. The advantages of the new HCSB materials include improved handling characteristics compared to MTA, such as being pre-mixed for direct dispensing into the root canal system, increased resistance to washout and reduced potential to cause discolouration [8–10]. Currently, there is minimal evidence available assessing outcomes of orthograde endodontic treatment following the use of HCSB putty material as a root end filling, despite prior clinical trials demonstrating success rates above 80% in surgical retrograde procedures with this material type [11, 12]. As a result of these documented advantages, a UK teaching hospital treatment protocol was adapted to use HCSB TotalFill BC Putty for endodontic apexification procedures. Therefore, this evaluation aimed to retrospectively assess the clinical and radiographic outcomes of the use of TotalFill BC Putty (TFBCP) for apexification of permanent maxillary teeth in children and adolescents.

## Materials and methods

### Study design

This retrospective evaluation included patients aged 16 or under, who underwent primary orthograde apexification in permanent teeth, between 2016 and 2023, using TotalFill BC Putty. Treatment was completed by paediatric staff and postgraduate endodontic trainees based at a dental teaching hospital in the UK. Table 1 summarises the inclusion and exclusion criteria.

**Table 1:** Summary of the inclusion and exclusion criteria.

Inclusion Criteria	Exclusion Criteria
Permanent teeth	Primary Teeth
Diagnosis of Pulp Necrosis	Diagnosis of Vital Pulp or Pulpitis
History of trauma leading to pulp necrosis	Caries leading to pulp necrosis
Age 16 or under	Age 17 and above
Primary orthograde endodontic treatment	Endodontic Retreatments, Pulpotomies or Regenerative Procedures
Treatment completed by hospital dentists or postgraduate endodontic trainees	Treatment completed by undergraduate students
Treatment performed under local anaesthetic or no anaesthetic.	Gutta Percha, MTA or any other material used as an apical plug for root end closure
Treatment performed under inhalation sedation (nitrous oxide).	Treatment under General Anaesthesia
TotalFill BC Putty used as an apical plug for root end closure	Patients included in any other trials.
Obturation completed with TotalFill BC putty only or TotalFill BC putty apical plug and Gutta Percha delivered with the warm vertical compaction technique	

Case identification was facilitated using the Carestream PACS electronic radiograph system (Carestream, New York, USA). A database of paediatric patients requiring periapical radiographs from January 2016 to June 2023 was generated. Radiographs were screened, and patients with a history of endodontic treatment were selected. Eligible patients’ clinical notes were then analysed to document case history and treatment details, which were recorded in a database. This project was registered with the clinical effectiveness department of the Royal Liverpool Dental Hospital as a service evaluation (Project Number 12204). As per Health Research Authority Decision Tool, no ethical review was required [13].

### Clinical and radiographic outcome assessment

Success was assessed radiographically using the periapical index (PAI) [14] and clinically, against the European Society of Endodontology quality guideline consensus report (Table 2) [15]. The signs and symptoms of infection assessed included tenderness to percussion, mobility, sinus tract formation and pain.

**Table 2:** European Society of Endodontology definitions of outcome [14].

Favourable	Clinical - No signs and symptoms of infection, no loss of function Radiological - normal PDL space around root
Uncertain	Clinical - No signs and symptoms of infection, no loss of function Radiological - no changes or reduction in size of lesion
Unfavourable	Clinical - Signs and symptoms of infection Radiological - lesion has increased in size or appeared subsequent to treatment. Signs of continuing root resorption.

**Fig. 1:**
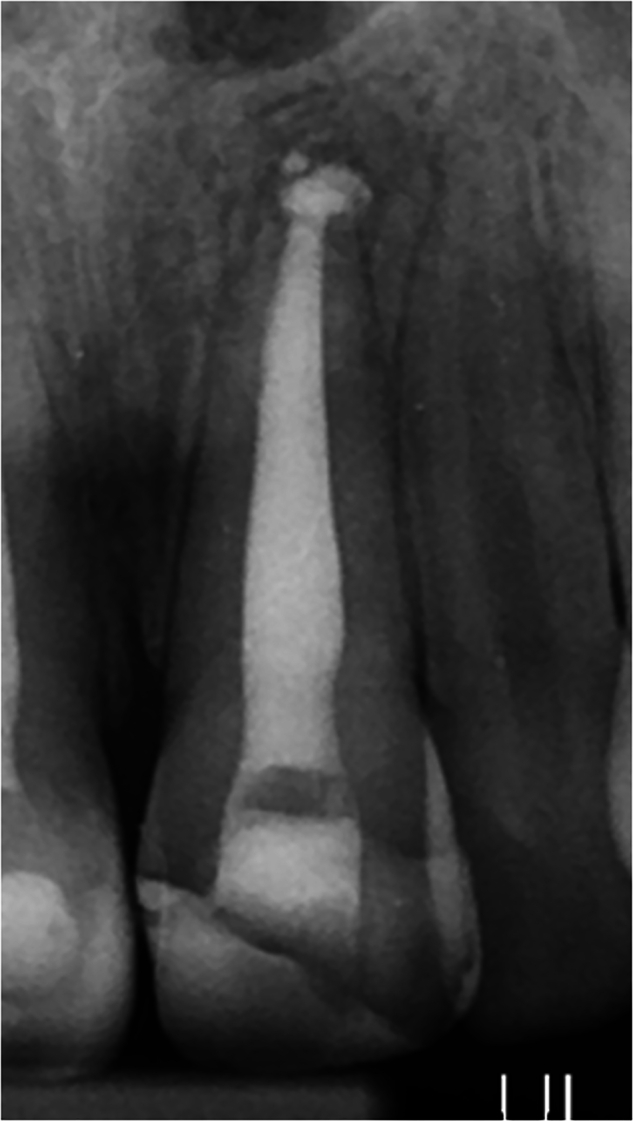
Periapical radiograph of previously treated maxillary incisor with small areas of TotalFill extrusion surrounded by ‘normal PDL’.

Radiographically, cases with small areas of TotalFill BC Putty plug extrusion were accepted as ‘normal PDL’ if minor bony changes were associated with the material only (Fig. 1). PAI scores of #1 or #2 with no signs or symptoms were considered as ‘Favourable’ outcome. ‘Uncertain’ outcomes were identified in asymptomatic cases scoring #3 or where a reduction of PAI was observed between #5 and #3. ‘Unfavourable’ outcome was assigned in cases with evidence of resorption/infection, a PAI score of #4 or #5 that remained unchanged at review, or cases where PAI score increased.

Successful treatment outcome was also loosely defined by the radiographic reduction in the size of the lesion as previously described by Ng et al. [16].

Baseline, post obturation and review digital radiographs were assessed by two independent examiners, both postgraduate dental core trainees. If there was disagreement over the outcome of individual cases, a discussion took place between the two examiners, and a consensus was reached. Both examiners were calibrated prior to the assessment of the included cases by evaluating 15 radiographs that were not included in this evaluation. Radiographs were evaluated under identical viewing conditions and cross referenced with case notes. If there was disagreement over the outcome of individual cases, a discussion took place and a consensus reached.

The Carestream system facilitated the use of measurement tools and contrast adjustment to aid assessment of the root filling and periapical changes.

### Variables affecting treatment

Table 3 shows the additional treatment variables which were recorded and entered into a database. Satisfactory technical quality of root filling was defined as a well condensed obturation, within 2 mm of the radiographic apex with no voids [17].

**Table 3:** List of recorded clinical variables.

Clinical Variables	
Type of tooth	Obturation method
Age at commencement of endodontic treatment	Staff Grade/ Experience
Mechanical preparation technique	Type of irrigant used
Type of trauma	Technical quality of root filling
Cooperation issues	Endodontic success outcomes
Number of treatment visits	Intervisit medicament

### Statistical analysis

Statistical analysis was completed using SPSS 29.0 statistical software (IBM, Armonk, NY, USA). The Kruskall-wallis test was used for analysis of secondary aims, to measure the effect of nominal and ordinal variables on the reduction of the PAI score. A ‘*P* value’ of <0.05 was assumed as statistically significant.

## Results

A total of 66 teeth from 57 patients were included in the evaluation. The mean number of visits to complete the endodontic treatment was 3.5 (range 2–10). Mean follow-up time was 14 months (range 2–61 months). For the purposes of this evaluation, the term ‘time of latest review’ represents the time elapsed since obturation, rather than the entire duration of treatment, which often involved multiple visits and interim calcium hydroxide dressings. As a result, the actual period of periapical healing may be longer than the post-obturation follow-up alone suggests.

The patients’ mean age was 10 years (range 7–16). Tables 4 and 5 summarise the relevant collected data.

**Table 4:** Summary of treatment and patient demographics.

Variables		
Type of tooth	Frequency	%
Maxillary Central Incisor (11, 21)	61	92.4
Maxillary Lateral Incisor (12, 22)	5	7.6
Age at commencement of endodontic treatment		
≥7– ≤ 10	36	54.5
≥11– ≤ 14	28	42.4
≥15- ≤16	2	3.1

**Table 5:** Distribution of trauma type by injury.

Trauma Injury Classification		
	Frequency	*%*
**Trauma to tooth only**		
Enamel-Dentine Fracture	20	30.3
Enamel-dentine Pulp Fracture	6	9.1
**Trauma to Periodontium only**		
Subluxation/ Concussion	1	1.5
Luxation	3	4.5
Extrusion	3	4.5
Intrusion	3	4.5
Avulsion	9	13.6
**Combined tooth and periodontal injuries**	8	12.1
**Unknown/Not recorded**	13	19.7

All teeth were diagnosed with pulp necrosis [18]. All teeth were treated under dental dam isolation and had an apical size greater or equal to 0.6 mm, as per definition of open-apex per Sarris et al. [19]. The apical size was verified by stainless steel K Files. Where appropriate, canals were mechanically prepared with stainless steel K Files by hand or with nickel titanium rotary instruments including ProTaper Gold (Dentsply Sirona, Ballaigues, Switzerland) and XP-Endo Finisher (FKG Dentaire. La-Chaux-de-Fonds, Switzerland) in accordance with the manufacturers’ instructions. All staff had prior training in rotary and hand instrumentation techniques. The irrigation protocol primarily involved 0.2% chlorhexidine, which was used in 94% of cases (*n* = 62). In a minority of cases, 1% sodium hypochlorite was used (3%, *n* = 2), while a combination of 1% sodium hypochlorite and 0.2% chlorhexidine was utilised in the remaining cases (3%, *n* = 2). Variations in irrigant selection were dependent on multi-visit treatment strategies and clinician preference. Working length was assessed with the aid of a periapical radiograph. All teeth were medicated with an intervisit medicament which included non-setting calcium hydroxide, Vitapex (Neo dental co., Tokyo, Japan) or double antibiotic paste. Obturation was performed only in asymptomatic teeth with a dry canal, which was the main determinant in the number of conducted treatment visits. A minimum of 4 mm of TotalFill BC Putty apical plug was placed with the aid of machtou hand pluggers (VDW, Munich, Germany) for compaction. The position and size of the plug was verified radiographically. The remaining coronal canal space was backfilled in the same appointment using either further TotalFill BC Putty or thermoplastic gutta percha (Elements gutta percha cartridge, Sybron Endo, Kerr Dental, Uxbridge, UK) with the additional use of zinc oxide eugenol based (Tubli-seal™, Kerr Dental, Uxbridge UK) or Tricalcium Silicate Cement-Based root canal sealer (TotalFill BC Sealer, FKG Dentaire, Switzerland). Access cavities were restored with a range of glass ionomer, resin modified glass ionomer or resin composite restorations. Base-line and review periapical radiographs were taken using a paralleling technique and photostimulable phosphor plates.

### Clinical and radiographic outcome assessment

**Fig. 2:**
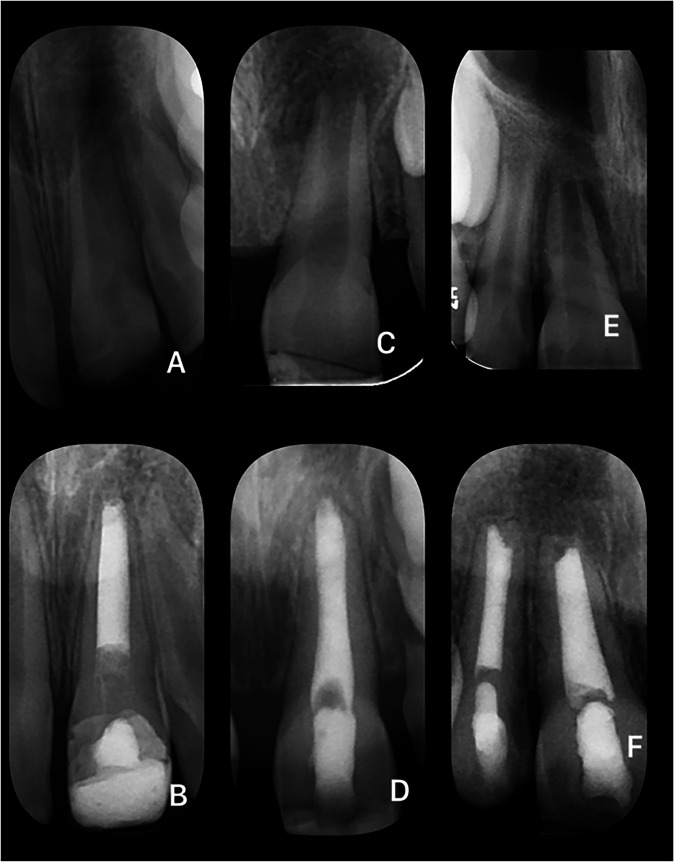
Pre- and post-operative radiographs of example cases with a “Favourable” outcome. **A** Maxillary central incisor in a patient between 5 and 10 years old with a history of enamel-dentine fracture (Pre-op). **B** The same tooth at 8 months post-treatment. **C** Maxillary central incisor in a patient between 5 and 10 years old with a history of avulsion (Pre-op). **D** The same tooth at 11 months post-treatment. **E** Maxillary central and lateral incisors in a patient between 5 and 10 years old with a history of avulsion and enamel-dentine fracture (Pre-op). **F** The same teeth at 7 and 18 months post-treatment.

**Fig. 3:**
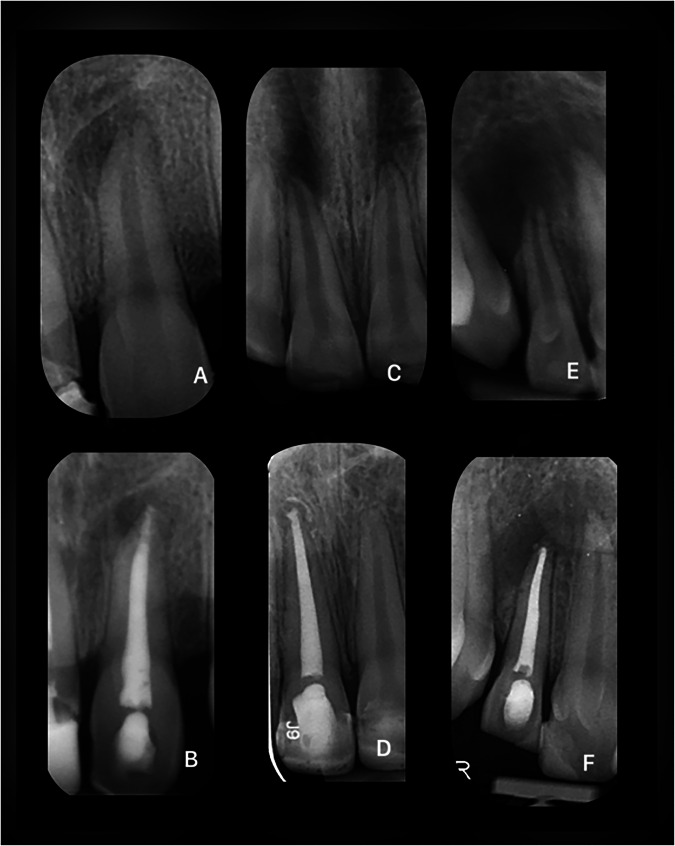
Pre- and post-operative radiographs of example cases with an “Uncertain” outcome. **A** Maxillary central incisor in an adolescent patient with a history of unknown trauma (Pre-op). **B** The same tooth at 4 months post-treatment. **C** Maxillary central incisor in a patient between 5 and 10 years old with a history of enamel-dentine fracture (Pre-op). **D** The same tooth at 11 months post-treatment. **E** Maxillary lateral incisor in an adolescent patient with a history of unknown trauma (Pre-op). **F** The same tooth at 15 months post-treatment.

**Fig. 4:**
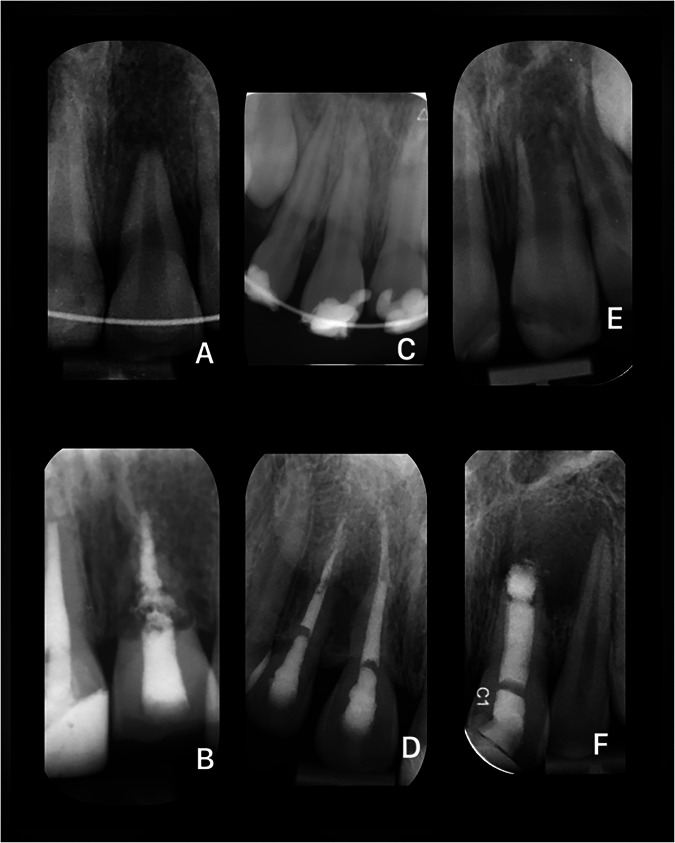
Pre- and post-operative radiographs of example cases with an “Unfavourable” outcome. **A** Maxillary central incisor in a patient between 10 and 15 years old with a history of avulsion (Pre-op). **B** The same tooth at 53 months post-treatment. **C** Maxillary central and lateral incisors in a patient between 10 and 15 years old with a history of avulsion (Pre-op). **D** The same teeth at 5 months post-treatment. **E** Maxillary central incisor in a patient between 5 and 10 years old with a history of enamel-dentine fracture (Pre-op). **F** The same tooth at 43 months post-treatment.

Success outcome at latest review was favourable in 48.5% (*n* = 32), uncertain in 36.4% (*n* = 24) and unfavourable in 15.1%(*n* = 10) of cases. In 84.9% (*n* = 56) of cases, there was evidence of complete or partial radiographic healing. Table 6 shows the summary of the observed pre and post operative PAI scores. Notably, there is a significant decrease in PAI scores at the time of the latest review compared to the initiation of treatment, indicating evidence of radiographic healing. Example radiographs of treated cases across all of the three outcomes are shown in Figs. 2–4.

**Table 6:** Summary of the pre and post OP PAI scores and the outcome.

Outcome Measures		
	Frequency	%
Pre-Op PAI		
1	0	0
2	2	3.0
3	10	15.2
4	22	33.3
5	32	48.5
Post Op PAI		
1	11	16.7
2	25	37.9
3	16	24.2
4	11	16.7
5	3	4.5
Outcome		
Favourable	32	48.5
Uncertain	24	36.4
Unfavourable	10	15.1

### Variables affecting treatment

Patient Cooperation issues and staff grade showed no statistical significance on the observed change in the PAI score (Table 7). The technical quality of the obturation demonstrated a statistically significant effect on the reduction in the PAI score. Further details on the remaining recorded clinical variables can be found in SI Table 1, available in the supplementary material section.

**Table 7:** Summary of dental treatment variables and their effects on the reduction of the PAI score.

Treatment Variables		
	Value	%
Technical Quality		
Unsatisfactory	5	7.6
Satisfactory	61	92.4
Kruskal-Wallis *p*-value	<0.01	
Staff Grade		
Dental Core Trainee Junior Grade	5	7.6
Paediatric Registrar/ Endodontic PG Student	46	69.7
Paediatric Consultant/Specialist	15	22.7
Kruskal-Wallis *p*-value	0.459	
Cooperation Issues		
Nil	45	68.2
Anxiety	15	22.7
Movement Issues	6	9.1
Kruskal-Wallis *p*-value	0.623	

### Analysis of cases with the unfavourable outcome

A total of 10 cases (15.2%) were deemed to have an unfavourable outcome due to clinical signs and symptoms of infection and/or radiological evidence of resorption or increase in lesion size. Avulsion was the most commonly recorded injury type, with 50% of cases (*n* = 5) presenting with this injury. The presence of obturation voids was noted in 40% (*n* = 4) and the obturation was greater than 2 mm from the radiographic apex in 20% (*n* = 2) of unfavourable cases. At the review appointment, resorption was recorded in 40% (*n* = 4) of cases, one case presented with mobility (10%) and one case was tender to percussion testing (10%). Furthermore, the PAI score increase was noted in two cases (20%) and the PAI score did not change from PAI 5 in three cases (30%) (Table 8).

**Table 8:** Summary of recorded clinical variables.

Treatment Variables		
	Value	%
Mechanical preparation technique		
None recorded	1	1.5
Hand	55	83.3
Rotary	10	15.2
Number of treatment visits		
Two	17	25.8
Three	27	40.9
Four	11	16.7
Five	6	9.1
Six	2	3.0
Seven	1	1.5
Eleven	1	1.5
Twelve	1	1.5
Obturation method		
Total Fill Plug + Warm Vertical Compaction Gutta Percha	38	57.6
Total Fill Complete	28	42.4
Type of irrigant used		
Chlorhexidine 0.2%	62	93.9
Sodium Hypochlorite 1%	2	3.0
NaOCl 1% and CHX 0.2%	2	3.0
Intervisit medicament		
CaOH	65	98.5
Double Antibiotic Paste and CaOH	1	1.5

## Discussion

This retrospective evaluation aimed to evaluate the clinical and radiographic outcomes of apexification in permanent maxillary teeth with pulpal necrosis with TFBCP. There is currently very limited evidence available regarding the outcomes of endodontic treatment utilising TFBCP or EndoSequence Root Repair Material (ESRRM) for orthograde endodontic apexification procedures. Recent study by Donnell and Kandiah reported a 92% success rate for apexification using TotalFill BC Putty in 25 cases of immature permanent incisors with open apices at a 12-month follow-up [20]. Due to the versatility of the TFBCP, several outcome studies have been published exploring the treatment outcomes of apical microsurgery procedures, pulpotomies and management of open apices. Success rate of 91.9% at 12-month follow up was reported by Taha et al. using TFBCP in full pulpotomy procedure in mature permanent molars presenting with irreversible pulpitis [8]. Similar outcomes of 92.4% with at least 6-month follow up were reported by Chan et al. using ESRRM in endodontic microsurgery procedures delivered by graduate endodontic residents [21]. A published dissertation by Sarnowski et al. has reported radiographic healing in 92% of 36 investigated cases with open apices with a median follow up of 18 months. The evaluation included a mixture of materials used for the apexification including ProRoot MTA, ProRoot White MTA, Neo-MTA and ESRRM with unknown number of cases of each material included [22]. Classically, MTA has been long established as the material of choice for apexification of immature apices with range of studies reporting success rates from 77% with an average of 12 month follow up to 96.9% with a mean follow up of 30 months [19, 23].

The PAI index based on the assessment of two-dimensional periapical radiograph was used in this study to assess the periapical status of the investigated teeth before endodontic treatment and at review following a period of healing [14]. It has been extensively validated through its use in previous studies, and has been shown to be a reliable tool for measuring radiographic presence of apical periodontitis [23, 24].

Although periapical radiographs are readily accessible and economically viable, they are not without limitations. These can include the potential superimposition of anatomical structures, which may impede the accurate detection and assessment of periapical lesions. Cone-beam computed tomography (CBCT) is frequently used to assess the presence of periapical periodontitis as it allows full visualisation of the apical anatomy in three dimensions and has been shown to have better diagnostic accuracy. However, CBCT has higher financial cost, and may result in a higher dose of ionising radiation to the patient especially for this paediatric patient cohort with an average age of 10 years. A study by Balasundaram et al. has compared the use of PA vs. CBCT radiographs in determination of the size of the periapical lesion and the proposed treatment and has found no statistical significance on the decision making regarding the treatment planning amongst the observers [25]. However, Patel et al. has shown that use of CBCT to diagnose and treatment plan Traumatic Dental Injuries (TDIs) in the maxillary anterior region, has resulted in enhanced diagnostic ability and improved confidence of diagnosis of the surveyed clinicians. Additionally, the CBCT has shown higher sensitivity and specificity and also improved the level of agreement amongst clinicians regarding management of the TDIs [26]. Therefore, the use of CBCT to assess the treatment outcomes should be considered in future prospective studies.

Interestingly, a study by Safi et al. compared healing outcomes following the use of MTA and ESRRM in endodontic microsurgery procedures and has shown that CBCT evaluation resulted in lower healing rates observed compared to PA radiographs [11]. This study has also reported that root canal filling quality influenced the success of the treatment which is also reflected in the findings of this study. This can be further linked to the publication by Ng et al., which has shown that presence of obturation within 2 mm of the radiographic apex with absence of voids was found to statistically significantly improve endodontic outcomes [17].

Analysis of other treatment variables did not find a statistical influence on the observed reduction of PAI score. Surprisingly, staff grade and experience was not shown to be statistically significant to influence the treatment outcomes. This could be due to the fact that more clinically challenging cases were referred to more experienced clinicians.

Analysis of the cases with the unfavourable outcome revealed a high prevalence (50%) of avulsion injuries. It is one of the most serious TDIs with high risk of complications including inflammatory and replacement resorption. Notably, all instances of recorded resorption in this evaluation were associated with the avulsion injury, therefore the authors speculate that the material used to treat these cases may have less to little influence on the outcome of treatment. The current dental trauma guidelines emphasise the importance of rapid reimplantation following the injury to keep the periodontal ligament cells viable, reducing the risk of infection or damage [27]. If precementum and cementoblast layers are damaged, macrophages, neutrophils and osteoclasts in the surrounding tissues can initiate the process of root resorption [28, 29]. These processes can occur very rapidly and lead to complications such loss of the affected teeth and risk of ankylosis, therefore regular clinical and radiographic follow up is indicated [27]. Inclusion of records of extra-oral conditions prior to reimplantation for avulsion cases should be considered in future studies. Variables such as extra-oral dry time, the type of storage medium (if any) used, and whether extensive physical cleaning was required prior to reimplantation, could provide valuable insights into the factors influencing treatment outcomes.

This service evaluation has multiple limitations which need to be taken into consideration when interpreting the results. The retrospective design is susceptible to selection bias, potentially resulting in a sample that may not be fully representative of all eligible cases. In some cases, follow-up reviews occurred as early as two months post-obturation, which may not provide sufficient time for radiographic healing to become evident. However, given that many teeth in this study underwent multi-visit treatment with interim calcium hydroxide dressings, healing may have already started before the final obturation. Nonetheless, this variability in follow-up timing presents a limitation, and future studies with standardised, longer-term reviews would help to better assess treatment outcomes.

Additional limitations of this study include a limited sample size and the lack of a power calculation. Whilst measures were taken to ensure all treated cases were identified, the absence of these calculations implies that the study might not have been adequately powered to detect significant differences or trends, particularly in the context of subgroup analyses. Furthermore, multiple cases which were deemed to have good term prognosis were immediately discharged following the completion of treatment and were not included in the evaluation. This exclusion of potentially successful cases could lead to an underrepresentation of positive outcomes, affecting the perceived endodontic treatment success rate. A critical limitation of study was the lack of the standardised treatment protocol across all cases. Due to the retrospective nature of this project, there were multiple variations in clinical practice, which included a range of operator experience, the type of endodontic sealer and definitive restorative materials used. Additionally, levels of magnification used by treating clinicians and the use of various types of file systems, introduce a considerable degree of heterogeneity to the treatment process. This lack of standardisation could have significant implications on the consistency and comparability of the treatment outcomes, thereby impacting the overall conclusions that can be drawn about the effectiveness and reliability of the treatment protocol. However, in contrast, the inclusion of multiple clinicians with various experience improves the generalisability of the findings. Lastly, the inclusion of patients aged between 7 to 16 years old in the evaluation introduces further significant heterogeneity in presenting root development stages, leading to notable variations in apical sizes across cases. This variability in root maturation may have an additional effect on the endodontic treatment outcomes therefore the findings need to interpreted with caution. Future research should focus on prospective, randomised controlled trials with more rigorous methodologies and standardised protocols to validate and extend these findings. This study did not include a cost-benefit analysis of TotalFill BC Putty compared to other materials. However, future research could explore its economic viability in clinical practice, particularly in paediatric endodontics. Additionally, considering that apexification with HCSBs is the standard for managing immature non-vital teeth, the development of a framework for study reporting is crucial. Such a framework would facilitate data analysis across centers and support the establishment of evidence-based recommendations.

## Conclusion

This evaluation demonstrated that TotalFill BC Putty is an effective material for apexification in immature non-vital permanent maxillary teeth, showing favourable clinical and radiographic outcomes. The findings highlight its potential as a reliable treatment option within the paediatric population. However, variability in treatment protocols and operator experience underscores the need for standardisation to enhance clinical outcomes and comparability. Given that this project was a service evaluation without a pre-determined power calculation, the conclusions drawn should be viewed within these limitations. Future research should incorporate structured methodologies and sample size calculations to provide stronger evidence.

## Supplementary information

Supplementary information

## Data Availability

The data supporting the findings of this study are available upon reasonable request from the corresponding author. Certain patient identifying details have been removed to protect confidentiality.
