# Supplementary material for: The outcomes of calcium silicate cement putty apical plugs in traumatised permanent maxillary teeth in paediatric patients: a retrospective evaluation
Source: BDJ Open. 2025 Apr 16;11:39. doi: 10.1038/s41405-025-00317-9 (PMC12003701; doi:10.1038/s41405-025-00317-9)
Supplement: Supplementary file 1 — Supplementary information [file 41405_2025_317_MOESM1_ESM.docx]

| Supplementary Table 1 Summary of Recorded Clinical Variables | | |
| --- | --- | --- |
| Treatment Variables | | |
|  | **Value** | **%** |
| Mechanical preparation technique |  |  |
| None recorded | 1 | 1.5 |
| Hand | 55 | 83.3 |
| Rotary | 10 | 15.2 |
| Number of treatment visits |  |  |
| Two | 17 | 25.8 |
| Three | 27 | 40.9 |
| Four | 11 | 16.7 |
| Five | 6 | 9.1 |
| Six | 2 | 3.0 |
| Seven | 1 | 1.5 |
| Eleven | 1 | 1.5 |
| Twelve | 1 | 1.5 |
| Obturation method |  |  |
| Total Fill Plug + Warm Vertical Compaction Gutta Percha | 38 | 57.6 |
| Total Fill Complete | 28 | 42.4 |
| Type of irrigant used |  |  |
| Chlorhexidine 0.2% | 62 | 93.9 |
| Sodium Hypochlorite 1% | 2 | 3.0 |
| NaOCl 1% and CHX 0.2% | 2 | 3.0 |
| Intervisit medicament |  |  |
| CaOH | 65 | 98.5 |
| Double Antibiotic Paste and CaOH | 1 | 1.5 |
